# A two-task predictor for discovering phase separation proteins and their undergoing mechanism

**DOI:** 10.1093/bib/bbae528

**Published:** 2024-10-21

**Authors:** Yetong Zhou, Shengming Zhou, Yue Bi, Quan Zou, Cangzhi Jia

**Affiliations:** School of Science, Dalian Maritime University, 1 Linghai Road, Dalian, 116026, China; College of Computer and Control Engineering, Northeast Forestry University, No. 26 Hexing Road, Xiangfang District, Harbin, 150040, China; College of Life Science, Northeast Forestry University, No. 26 Hexing Road, Xiangfang District, Harbin, 150040, China; Department of Biochemistry and Molecular Biology, Monash University, Melbourne, Victora 3800, Australia; Institute of Fundamental and Frontier Sciences, University of Electronic Science and Technology of China, No. 2006, Xiyuan Ave, West Hi-Tech Zone, Chengdu, 611731, China; School of Science, Dalian Maritime University, 1 Linghai Road, Dalian, 116026, China

**Keywords:** phase separation, self-assembly, partner-dependent, sequence analysis, deep learning

## Abstract

Liquid–liquid phase separation (LLPS) is one of the mechanisms mediating the compartmentalization of macromolecules (proteins and nucleic acids) in cells, forming biomolecular condensates or membraneless organelles. Consequently, the systematic identification of potential LLPS proteins is crucial for understanding the phase separation process and its biological mechanisms. A two-task predictor, Opt_PredLLPS, was developed to discover potential phase separation proteins and further evaluate their mechanism. The first task model of Opt_PredLLPS combines a convolutional neural network (CNN) and bidirectional long short-term memory (BiLSTM) through a fully connected layer, where the CNN utilizes evolutionary information features as input, and BiLSTM utilizes multimodal features as input. If a protein is predicted to be an LLPS protein, it is input into the second task model to predict whether this protein needs to interact with its partners to undergo LLPS. The second task model employs the XGBoost classification algorithm and 37 physicochemical properties following a three-step feature selection. The effectiveness of the model was validated on multiple benchmark datasets, and *in silico* saturation mutagenesis was used to identify regions that play a key role in phase separation. These findings may assist future research on the LLPS mechanism and the discovery of potential phase separation proteins.

## Introduction

Liquid–liquid phase separation (LLPS) is the process by which membraneless organelles are assembled into droplets through the interactions of various proteins, DNA, or RNA [1, 2]. It has been recognized as a universal mechanism for the formation of biomolecular condensates in cells, regulating biological processes such as gene expression and signal transduction [3–5]. Increasing evidence also suggests that abnormal assembly of membraneless organelles may lead to neurodegenerative diseases, tumors, aging, and other diseases [6, 7]. This phenomenon has generated significant interest in the field of drug discovery, with LLPS proteins considered potential therapeutic targets for cancer and neurodegenerative diseases [8, 9]. To support the development of drugs targeting these diseases, it is essential to discover more disease-related biomolecular aggregates and proteins and explore the physicochemical properties of aggregates as well as the conditions and mechanisms driving their assembly [10]. This is crucial for the discovery of new drug targets and the development of innovative drugs [11]. Despite ongoing research on the principles and applications of biomolecular condensates, high-throughput screening methods for identifying endogenous-phase-separated proteins are still lacking. With the advancement of deep learning, the prediction and simulation of LLPS proteins are viewed as powerful tools for understanding protein folding mechanisms and folding protein functions, providing theoretical predictions for experimental research, and helping elucidate protein molecular mechanisms [12].

Researchers have proposed numerous computational prediction methods for forecasting phase separation proteins [13]. Previous prediction approaches were primarily based on analytical methods, such as structural features or amino acid propensities [14–18]. Leveraging the LLPSDB [19], PhaSePro [20], PhaSepDB [21], and DrLLPS [22] databases, sequence-based LLPS protein prediction tools trained on large datasets have been widely proposed. DeePhase is an integrated model whose predictions for each sequence are set to be the average prediction made by the two submodels. One submodel utilizes word2vec encoding, while the other utilizes handcrafted features including sequence length; hydrophobicity; Shannon entropy; the fraction of the sequence identified to be part of the intrinsically disordered regions (IDRs) and low-complexity regions (LCRs); and the fraction of polar, aromatic, and cationic amino acid residues within the LCRs [23]. Based on the LLPSDB database, Chun *et al.* proposed PSPredictor by combining word2vec and gradient boosting decision trees (GBDTs) to predict scaffold phase separation (PS) proteins [24]. PhaSePred is the first predictor of self-assembling proteins (PS-Self) and partner-dependent proteins (PS-Part), respectively, and the phase separation behavior of proteins was experimentally verified *in vitro* [25]. Based on PS proteins sourced from the PhaSepDBV2.1, LLPSDBV2.0, and PhaSePro databases, PredLLPS_PSSM was established to predict PS, PS-Self, and PS-Part proteins using a position-specific scoring matrix (PSSM) within a deep learning framework [26]. MolPhase, employing 39 physicochemical characteristics and the GBDT classification algorithm, predicts phase-separated proteins and indicates that phytobacterial type III effectors are highly prone to homotypic PS [27]. PSPHunter utilizes a variety of features, including amino acid composition, evolutionary conservation, predicted functional site annotations, word embedding vectors, protein annotation information, and network properties, for predicting phase separating proteins and key residues to enhance its application and biological discovery [28]. Specifically, Ahmed *et al.* introduced the first predictor for the identification of RNA-dependent LLPS proteins based on sequence information and the random forest (RF) classification algorithm [29]. Despite the superior performance of sequence-based LLPS protein prediction tools, several issues remain unaddressed. Both DeePhase and PSPredictor, constructed solely on PS proteins from the LLPSDB, require updates to include LLPS proteins from other databases. Although recent tools like MolPhase and PSPHunter have demonstrated good performance, they are limited to distinguishing scaffold proteins as phase-separated proteins, which restricts their application scope. Thus, there is a need for a comprehensive predictor capable of not only determining whether a protein is an LLPS protein but also distinguishing between PS-Self and PS-Part proteins.

To address the deficiencies in our previous work, PredLLPS_PSSM, we have made several improvements in various aspects. In addition to the existing data on PredLLPS_PSSM, scaffold proteins from DrLLPS and artificially synthesized PS proteins from the LLPSDB were collected. Second, a hidden Markov model (HMM) containing homologous relationship information between proteins is combined with the PSSM to further characterize the similarity information or evolutionary information. Third, the physical and chemical properties crucial for the formation of PS are taken into consideration. Additionally, for the first time, a special model was established to distinguish between PS-Self and PS-Part proteins. More specifically, a two-task predictor named Opt_PredLLPS (optimization of PredLLPS_PSSM) was developed to discover phase separation proteins and determine whether they belong to the PS-Self or PS-Part categories. The first task model of Opt_PredLLPS integrates a CNN and BiLSTM through a fully connected layer, where the CNN utilizes evolutionary information features as input, and BiLSTM uses multimodal features as input. If a protein is predicted to be an LLPS protein, it is processed by the second task. The second task model of Opt_PredLLPS uses the XGBoost classification algorithm and 37 physicochemical properties following a three-step feature selection process. The model's effectiveness is confirmed across various benchmark datasets, while *in silico* saturation mutagenesis (ISM) is utilized to pinpoint regions essential for phase separation.

## Materials and methods

**Figure 1:**
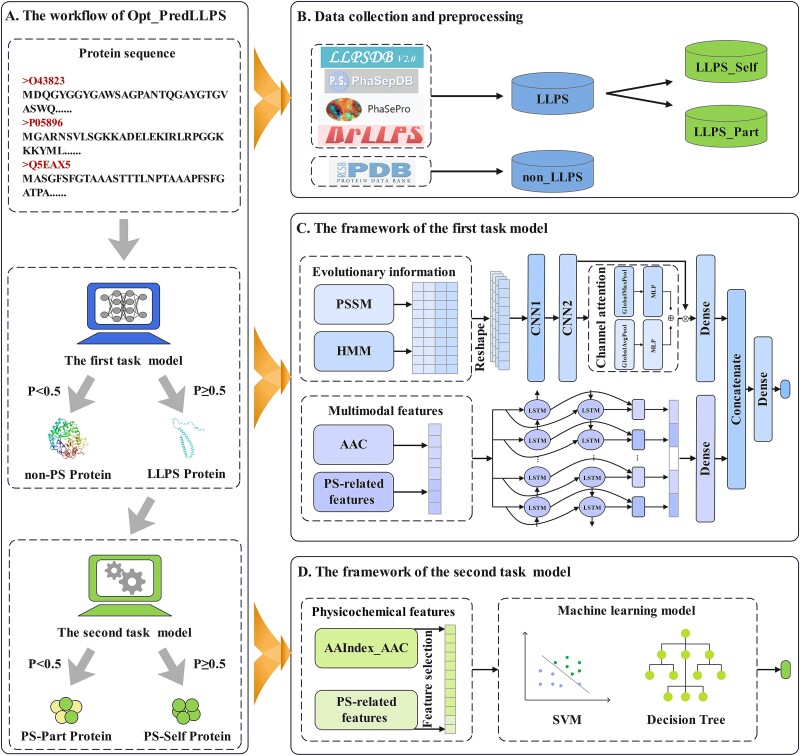
The overall framework of Opt_PredLLPS. (A) The workflow of Opt_PredLLPS. (B) Data collection and preprocessing. (C) The framework of the first task model. (D) The framework of the second task model.

As shown in Fig. 1, the structure of the study is segmented into four key components: the workflow of Opt_PredLLPS, data collection and preprocessing, the framework of the first task model and the framework of the second task model. Initially, for any given protein sequence, Opt_PredLLPS predicts whether the protein undergoes LLPS. If identified as an LLPS protein, the system further determines whether it is a PS-Self or PS-Part protein.

### Data collection and preprocessing

We collected the LLPS proteins from the latest databases, namely, LLPSDB2.0 [19], PhaSePro [20], PhaSepDB2.1 [21], and DrLLPS [22], which are built based on the literature on phase separation proteins or membraneless organelle (MLO) components. After filtering out protein sequences with invalid amino acids and lengths <50 or >5000, 3969 LLPS proteins were ultimately retained from these four databases. High similarity of proteins can cause overfitting of the model; thus, we removed those protein sequences with >40% similarity using CD-HIT [30, 31], resulting in 798 LLPS proteins as our positive benchmark dataset. To construct the negative benchmark dataset, we processed the PDB^*^ dataset, which was initially constructed by DeePhase [23]. We removed the protein sequences recorded in the above four PS databases from the PDB^*^ and those proteins with a similarity >40% using CD-HIT. Finally, we selected the same number of 798 protein sequences from the PDB^*^ dataset as from the non_LLPS (negative datasets). In addition, we split the LLPS dataset into an LLPS_Self dataset containing 285 PS-Self proteins and an LLPS_Part dataset containing 496 PS-Part proteins. In particular, 17 confusing proteins were omitted from our study. Moreover, the PS-Self (SaPS), the PS-Part (PdPS), and two independent test datasets (SaPS_Test and PdPS_Test), first used in PhaSePred [25], and two independent test datasets (Ind_Test_I and Ind_Test_II) built in PredLLPS_PSSM [26] are used to make an objective and fair comparison with existing predictors. All the datasets used in this work are provided in https://github.com/Zhou-Yetong/Opt_PredLLPS, and their summary statistics is listed in Table 1.

**Table 1:** Statistics of the datasets used in this study.

Dataset	Model	Positive samples	Negative samples
LLPS / non_LLPS	Opt_PredLLPS	798	798
LLPS_Self / LLPS_Part	Opt_PredLLPS	285	496
SaPS	PhaSePred	128	128
PdPS	PhaSePred	214	214
SaPS_Test	PhaSePred	72	216
PdPS_Test	PhaSePred	111	333
Ind_Test_I	PredLLPS_PSSM	48	144
Ind_Test_II	PredLLPS_PSSM	140	420

### Feature extraction

#### Amino acid composition

The amino acid composition (AAC) is expressed as the frequency of occurrence of amino acids in a protein sequence. The frequency of the 20 natural amino acids can be calculated as follows:


(1) \begin{equation*} f(x)=\frac{N(x)}{N},x\in \left\{A,C,D,...,Y\right\}, \end{equation*}

where *N* (*t*) is the number of amino acid types *t*, and *N* is the length of the protein sequence [32].

#### Physical and chemical property features

The amino acid indices (AAIndex) database [33], which encompasses a variety of physicochemical properties of amino acids and amino acid pairs (https://www.genome.jp/aaindex/), was utilized in this study. After the removal of physicochemical properties labeled as “NA,” 531 physicochemical properties were retained for further encoding analysis. To address the challenge of sequences of unequal lengths, a novel feature encoding method combining the frequency and physicochemical properties of amino acids, termed AAIndex_AAC, was proposed.


(2) \begin{equation*} {F}_j=\sum \limits_{i=1}^{20}\left(f\left({x}_i\right)\times{A}_{ij}\right),j\in \left\{1,2,3,...,531\right\}. \end{equation*}

where $f\left({x}_i\right)$ is the amino acid frequency of amino acid type *i*, ${A}_{ij}$ is the *j*-th physicochemical property of the *i*-th amino acid, and, finally, each protein sequence is represented by a 531-dimensional vector.

#### PS-related features

Studies have indicated that LCRs and large IDRs can phase separate under physiological conditions [1, 34]. Therefore, potentially disordered regions are generated utilizing ESpritz [35]; the lengths of the longest PS IDR and PS potential are calculated using ParSe2.0 [36]; the LCR score is obtained using StatSEG (https://github.com/jszym/StatSEG); and prion-like domain score (PLD-forming), granule propensity, and $\mathrm{\pi}$-$\mathrm{\pi}$ interaction are generated utilizing PLAAC, catGRANULE, and PScore, respectively [14–16]. In addition, the charge pattern and hydrophobicity can also affect the phase separation of proteins [37, 38]. The fraction of charged residues (FCR), net charge per residue (NCPR), Kappa, Omega, and polyproline II (PPII) propensity were obtained from LocalCIDER [39]. Furthermore, the hydrophobicity of the protein sequence was calculated using the Kyte and Doolittle hydropathy scale [40]. Finally, the Shannon entropy of each sequence is calculated as:


(3) \begin{equation*} H(X)=-\sum \limits_{i=1}^{N=20}{p}_i{\log}_2{p}_i, \end{equation*}

where ${p}_i$ is the frequency of amino acids [23].

#### Evolutionary information features

The position-specific score matrix (PSSM), an evolutionary information generated by multiple sequence alignments, contains a broad spectrum of information and has been successfully applied in protein prediction [41]. The PSSM feature vector is *L* × 20 dimensional, where *L* represents the length of the protein sequence and 20 corresponds to the number of amino acids. The POSSUM server was utilized to obtain PSSM feature files for protein sequences [42].

HMMs also contain homologous relationship information between proteins. We used HHblits with the parameter “-n 3 -e 0.01” to search the query sequence in the Uniclust30 database to obtain the HMM profile [43]. The first 20 columns of the HMM were obtained by $-1000\times{\log}_2^{h_{ij}^{\hbox{'}}}$, where ${h}_{ij}^{\hbox{'}}$ is the frequency of the amino acid matching state. The specific formula is as follows:


(4) \begin{equation*} {h}_{ij}^{\hbox{'}}={2}^{-0.001\times{h}_{ij}}\left(i\in \left\{1,2,\cdots, L\right\}1,j\in \left\{1,2,\cdots, 20\right\}\right). \end{equation*}

Furthermore, the amino acid matching state frequency serves as the input feature for our model. The HMM feature vector is also *L* × 20 dimensional, mirroring the dimensions of the PSSM feature vector [44]. A significant challenge in constructing feature vectors based on evolutionary information is the variability in protein lengths. To overcome this, a standard length of 5000 was established, and sequences shorter than this length were extended by appending zeros at the end.

### Model architectures

As shown in Fig. 1, we constructed a two-layer task classification model aimed at identifying the LLPS, PS-Part, and PS-Self proteins. According to the difference in the number of samples, we constructed deep learning models and traditional machine learning models, respectively.

The CNN utilized in this study consists of a convolutional layer, pooling layer, and fully connected layer. The convolutional layer and pooling layer are employed for feature extraction and dimensionality reduction, while the fully connected layer facilitates the final classification task [45, 46]. Long short-term memory (LSTM) networks are capable of maintaining the continuity and stability of information in long sequential data [47]. As an enhancement to LSTM, bidirectional LSTM (BiLSTM) is designed to better capture sequence context information [48]. An integrated deep learning model, combining CNN and BiLSTM, was constructed with three types of features as inputs to identify LLPS proteins. The deep learning training process was conducted using the keras package in Python.

Due to the limited number of PS-Self and PS-Part proteins, we evaluated a series of traditional machine learning models, including decision tree–based classifiers [extreme gradient boosting (XGBoost), RF, light gradient boosting machine (LightGBM), GBDT, and extremely randomized trees (ExtraTrees)] and support vector machine (SVM). These models are executed in scikit-learn using default parameters to avoid overfitting [49].

### Model evaluation

Seven metrics are adopted to measure our prediction model that include sensitivity (Sn), specificity (Sp), accuracy (Acc), Matthews correlation coefficient (MCC), precision (Pre), F1-score, and the area under the receiver operating characteristic curve (AUC). These formulas are given as follows:


(5) \begin{align*} \left\{\begin{array}{l} Sn=\frac{TP}{TP+ FN}\kern16em \\[4pt]Sp=\frac{TN}{TN+ FP}\kern16em \\[4pt]Acc=\frac{TP+ TN}{TP+ TN+ FP+ FN}\kern11em \\[4pt]MCC=\frac{TP\times TN- FP\times FN}{\sqrt{\left( TP+ FP\right)\left( TP+ FN\right)\left( TN+ FP\right)\left( TN+ FN\right)}}\kern0.75em \\[4pt]Pre=\frac{TP}{TP+ FP}\kern15.5em \\[4pt]F1- score=2\times \frac{Pre\times Sn}{Pre+ Sn}\kern11.5em \end{array}\right. \end{align*}

where TP, TN, FP, and FN represent the number of true positives, true negatives, false positives, and false negatives, respectively.

## Results and discussion

### Analysis of protein sequence properties

**Figure 2:**
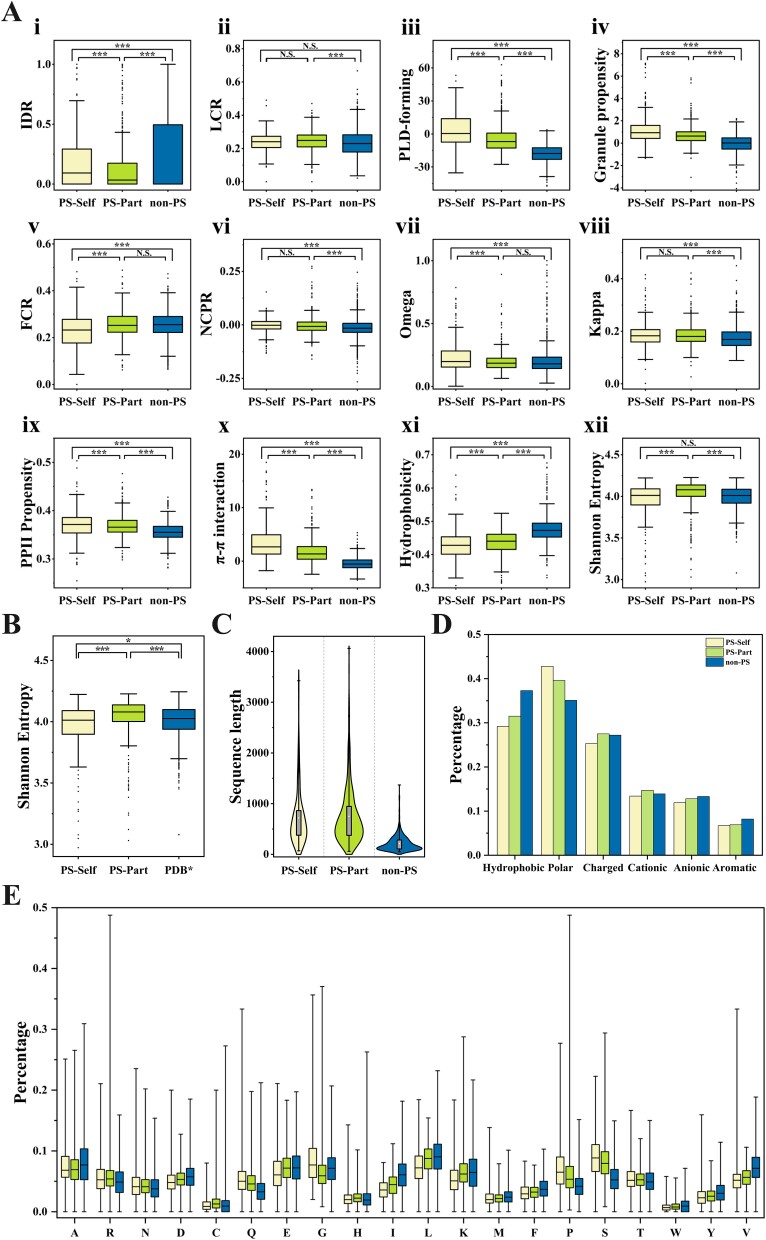
Sequence analysis of PS-Self, PS-Part, and non-PS proteins. (A) Comparison of 12 PS-related features. (B) Shannon entropy was compared between PS-Self proteins, PS-Part proteins, and proteins in the PDB^*^. (C) Violin plot of the sequence length distribution. (D) Proportion analysis of five different amino acid compositions in protein sequences. (E) Comparison of the percentages of amino acids. Symbols denote *P*-values of the Mann–Whitney U test (two-sided). Nonsignificant (N.S.): *P* > .05, ^*^P < .05, ^**^P < .01, ^***^P < .001.

The influence of amino acid composition and physicochemical properties on the undergo of PS was investigated by analyzing the average length and 12 physical and chemical properties (mentioned in Materials and Methods) of the PS-Self, PS-Part, and non-PS proteins. Significant differences in most features (Fig. 2A) were observed between the LLPS_Self dataset, the LLPS_Part dataset, and the non_LLPS dataset. Phase-separated proteins had relatively higher IDR, LCR, PLD-forming, and Granule propensity (Figure 2Ai–iv), which is consistent with previous conclusions [25]. Phase-separated proteins usually contain IDRs and LCRs. The flexible conformations and a large number of similar residues in IDRs and LCRs meet the requirements of LLPS [50]. Surprisingly, the FCR (Figure 2Av) of PS-Self proteins was significantly lower than that of PS-Part and non-PS proteins, while the FCR of PS-Part and non-PS proteins was not different. For NCPR (Figure 2Avi), the values of both the PS-Self and PS-Part proteins are close to zero and are slightly higher than those of the non-PS proteins [27, 51]. The higher Kappa and Omega values (Figure 2Avii–vii) in PS-Self suggest the formation of more localized blocks [27, 52, 53]. In contrast, there was a significant difference in the Kappa value and no significant difference in the Omega value between the PS-Part and non-PS proteins. Regarding PPII propensity and $\mathrm{\pi}$-$\mathrm{\pi}$ interaction, there were significant differences among the PS-Self, PS-Part, and non-PS proteins (Figure 2Aix–x). In general, the inter- and intramolecular interactions in PS-Self are the most obvious, which may be the reason why phase separation can occur without the participation of other macromolecules. The hydrophobic values of the non_LLPS dataset were higher than those of the two types of LLPS protein datasets, and this result was consistent with the results of hydrophobic amino acid analyses (Figure 2Axi), indicating that hydrophilic amino acids were enriched in the LLPS proteins. Interestingly, PS-Part proteins exhibited the highest Shannon entropy (Figure 2Axii), and there was no significant difference in Shannon entropy between PS-Self and non-PS proteins. The results for PS-Self proteins are not quite consistent with [27]. We speculate that this may be caused by the limited number of non-PS proteins. The Shannon entropy was further compared between PS-Self proteins and proteins in the PDB^*^ and showed certain differences, as shown by the *P*-value in Fig. 2B.

In addition, we inspected the sequence length, the composition of six groups of AAs, and the composition of 20 AAs. As shown in Fig. 2C, the average length of the PS proteins was longer than that of the structural proteins (non-PS), and the PS-Part proteins contained the longest sequences. The lengths of the PS proteins were mainly distributed in the range of 50–1000 aa, and the average lengths of the PS-Self proteins and PS-Part proteins were 719 and 750 aa, respectively. The length of non-PS proteins is mainly distributed in the range of 50–500 aa, and their average length is 213 aa. Overall, the length of non-PS proteins is shorter than that of PS proteins.

In Fig. 2D, both PS-Self and PS-Part proteins exhibited similarities in trends for Hydrophobic, Polar, Anionic, and Aromatic amino acids and displayed different trends for Charged and Cationic amino acids. Specifically, PS-Self and PS-Part proteins are rich in Polar amino acids, while they are less prevalent in Hydrophobic, Anionic, and Aromatic AAs. In contrast, fewer PS-Self proteins contain Charged and Cationic AAs, whereas PS-Part proteins are rich in these two groups of amino acids. Regarding the composition of amino acids in Fig. 2E, the glycine or proline is prevalent in PS proteins that are not conducive to the formation of normal secondary structures as indicated in [54].

In summary, PS-Self and PS-Part proteins exhibit the same trend in terms of most physicochemical properties but exhibit differences in Shannon entropy and the composition of charged and cationic AAs. The reasons for the significant differences observed between PS-Self and PS-Part proteins remain unclear, necessitating separate consideration of these two types of phase separation proteins in further research.

### The overall framework and performance of Opt_PredLLPS

Opt_PredLLPS is a two-task classification model aimed at identifying LLPS, PS-Part, and PS-Self proteins. The first task classification model comprises two critical components: evolutionary information features (PSSM and HMM) and multimodal features (AAC and PS-related features), respectively. The evolutionary information features are fed into a CNN equipped with a channel attention mechanism, which selectively enhances relevant information by weighting each channel of the feature map. Concurrently, multimodal features, including AAC and seven PS-related features (IDR, the length of the longest PS IDR, PS potential, PLD-forming, granule propensity, hydrophobicity, and Shannon entropy), are processed by a BiLSTM that extracts long-distance dependencies and implicit relationships between protein sequences. The outputs from both branches are then concatenated through a fully connected layer with a sigmoid activation function. The CNN module encompasses three sets of parameters: CNN1_filters $\in$ [16, 32, 64], CNN2_filters $\in$ [16, 32, 64], and dense_units $\in$ [8, 16, 32]. The BiLSTM module considers two sets of parameters: BiLSTM_units $\in$ [16, 32, 64] and dense_units $\in$ [8, 16, 32]. This configuration results in 243 possible combinations, with their 10-fold cross-validation results detailed in Tables S1–S5. The best AUC of 0.971 was reached with the optimal parameter combination [64, 32, 32, 32, 8].

**Figure 3:**
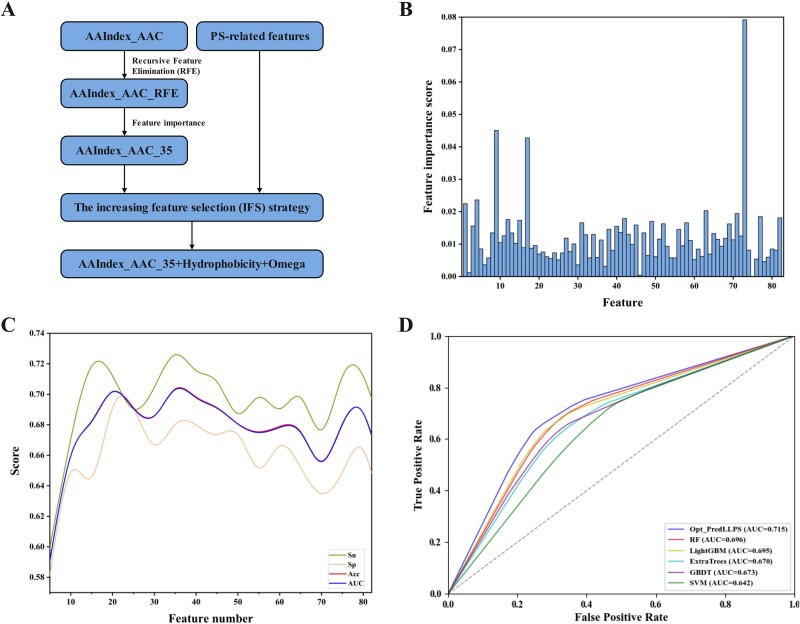
Feature and classifier selection in the process of model construction. (A) The process of feature selection. (B) The feature importance score of the AAIndex_AAC_RFE feature. (C) The performance of the model under different feature combinations. (D) 10-fold cross-validation results of different classifiers.

The second task model is designed to further identify PS-Part and PS-Self proteins. To the best of our knowledge, no computational tools have been built that specifically recognize these two types of proteins. A balanced training dataset was constructed by randomly selecting 285 PS-Part proteins from the LLPS_Part dataset and combining them with 285 PS-Self proteins. Given the limited sample size, only handcrafted features and traditional machine learning methods were employed. For the high dimensionality of AAC, AAIndex_AAC, and PS-related features, we adopted a three-step feature selection approach to find the optimal combination of features as shown in Fig. 3A. Initially, XGBoost was used to evaluate the performance of each feature type across a 10-fold cross-validation. As shown in Table 2, the model based on the AAC feature had an AUC of 0.614, the model based on PS-related features had an AUC of 0.615, and the model based on the AAIndex_AAC feature had an AUC of 0.640. The 531-dimensional AAIndex_AAC feature may contain redundant information and affect the model’s performance [55]. Therefore, we applied recursive feature elimination (RFE) integrated with the ExtraTrees algorithm to eliminate the redundant information of AAIndex_AAC [56]. When the number of dimensions is reduced to 82, the AUC increases from 0.640 to 0.682, with the highest value among the four feature-based models. Despite reducing the feature dimensions to 82, further refinement was necessary as these dimensions still exceeded the 20 and 12 dimensions of other features. Consequently, XGBoost was employed to assess the importance of the 82 features of AAIndex_AAC, defined by the frequency a feature divides the data, i.e. the number of times selected as weak classifier. The “Feature_importances” from the scikit-learn package calculated the importance scores for the AAIndex_AAC_RFE features (Fig. 3B), and features were ranked accordingly. The step size is set to 5 to find the feature subset with the best model performance. From Fig. 3C, it can be observed that the Acc and AUC curves overlap and both reach their peak at feature number 35, with an AUC value of 0.703. The optimal feature subset with feature number 35, named AAIndex_AAC_35, not only reduces the feature size but also improves the overall performance of the model. To further enhance the model, the increasing feature selection (IFS) strategy was utilized to incrementally add features to AAIndex_AAC_35. The detailed performance results for the IFS are given in the Supplementary Table S6. The model showed significant improvement when AAIndex_AAC_35, hydrophobicity, and Omega were incorporated, achieving an Sn of 0.709, an Sp of 0.722, and an AUC of 0.715, respectively, as detailed in Table 3. According to the classification types given in AAIndex [33], the diverse properties have been organized into six categories: alpha and turn propensities, beta propensity, composition, hydrophobicity, physicochemical properties, and other properties. Following the established criteria (Table S7), we identified 14 properties related to hydrophobicity from the 37 selected. Additionally, there were 9 properties associated with alpha and turn propensities, along with properties linked to beta propensity. Only 1 property pertained to composition, while 2 were related to physicochemical characteristics, and 4 were categorized as other properties. Furthermore, 7 properties remained ambiguous regarding their classification. The analysis results indicate that hydrophobicity and protein structure play a critical role in distinguishing between PS-Self and PS-Part proteins, aligning with the observations presented in Fig. 2. These findings enhance our understanding of phase separation proteins and facilitate the identification of potential PS-Self proteins.

**Table 2:** The 10-fold cross-validation results of different features.

Feature	Feature dimension	Sn	Sp	Acc	MCC	AUC
AAC	20	0.596	0.631	0.614	0.228	0.614
PS-related features	12	0.621	0.610	0.616	0.232	0.615
AAIndex_AAC	531	0.639	0.642	0.640	0.283	0.640
AAIndex_AAC_RFE	82	0.701	0.663	0.683	0.366	0.682

**Table 3:** The best performance of AAIndex_AAC_35 combined with other features through IFS.

Feature	Sn	Sp	Acc	MCC	AUC
AAIndex_AAC_35	0.726	0.680	0.704	0.409	0.703
AAIndex_AAC_35 + Hydrophobicity	0.740	0.684	0.712	0.426	0.712
AAIndex_AAC_35 + Hydrophobicity + Omega	0.709	0.722	0.716	0.432	0.715

In addition, we tested the classification performance of RF, LightGBM, ExtraTrees, GBDT, and SVM. For each classification algorithm, we used grid search for automatic parameter tuning and hyperparameter optimization to find the most suitable model. The parameter selection process and prediction results are detailed in Tables S8 and S9 and illustrated in Fig. 3D. As demonstrated in Fig. 3D and Table S9, the XGBoost model attained an AUC of 0.715, significantly outperforming other classification algorithms. In comparison, RF recorded the second highest AUC of 0.696 for identifying PS-Self and PS-Part proteins. Conversely, the SVM model exhibited the lowest AUC of 0.642, likely attributable to the limited sample size.

### Superiority of feature information fusion

**Figure 4:**
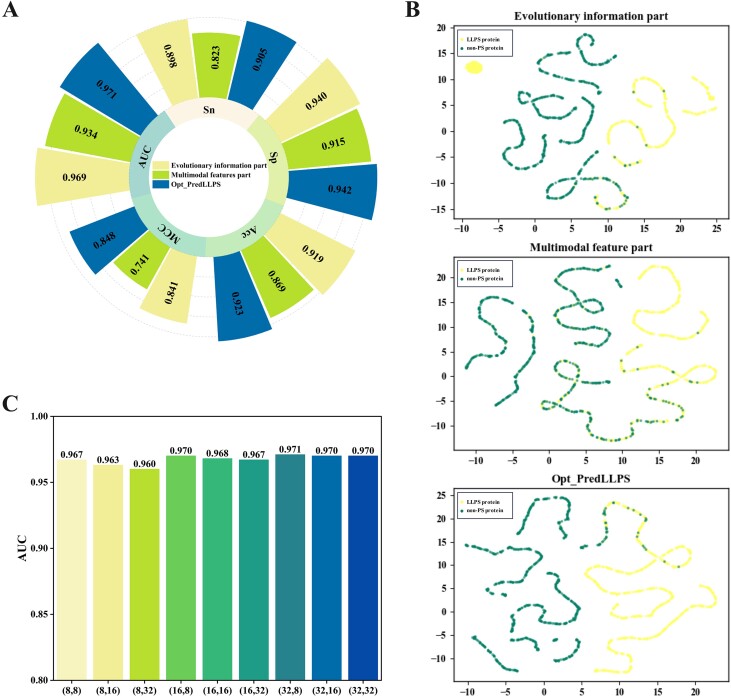
Ablation analysis of Opt_PredLLPS. (A) 10-fold cross-validation results of different models. (B) Visualization of different models in 2D space. (C) The AUC values of different feature combinations.

An ablation analysis was performed to investigate the effectiveness of each part of the Opt_PredLLPS. The evaluation involved the performance when using only the evolutionary information part and only the multimodal feature part. As illustrated in Fig. 4A, the results from 10-fold cross-validation indicated that the evolutionary information part achieved an Acc of 0.919 and an MCC of 0.841, which are 0.004 and 0.007 lower than those of Opt_PredLLPS, respectively. In contrast, the multimodal feature part achieved an Acc of 0.869 and an MCC of 0.741. To visually display the above results, we utilized the widely recognized visualization technique known as uniform manifold approximation and projection, commonly applied in bioinformatics. As shown in Fig. 4B, LLPS proteins and non-PS proteins are depicted as points of distinct colors within a 2D embedding space. However, many LLPS and non-PS proteins remain intermixed in the multimodal feature embedding graph, making them challenging to differentiate. Notably, aside from a few outliers, the evolutionary information part and Opt_PredLLPS successfully delineate the two protein categories. Specifically, LLPS proteins exhibit a more concentrated distribution in Opt_PredLLPS, demonstrating superior separation efficacy. These analyses demonstrated that the evolutionary information part is crucial within the entire architecture and that the integration of two components maximizes their advantages and minimizes the risk of overfitting. Moreover, different combinations of feature dimensions for the two output parts were meticulously evaluated. As shown in Fig. 4C and Table 4, the best AUC of 0.971 and Sn of 0.905 were recorded when the output feature dimension of the first part was 32 and that of the second part was 8. Conversely, the best Sp of 0.954, Acc of 0.925, and MCC of 0.852 were achieved when both the first and second parts had an output feature dimension of 8. Given the pivotal role of PS protein discovery in experiments, the feature combination (32, 8) with the highest Sn was selected.

**Table 4:** The performance of different feature combinations of the two output parts of Opt_PredLLPS.

Feature combination	Sn	Sp	Acc	MCC	AUC
(8,8)	0.896	0.954	0.925	0.852	0.967
(8,16)	0.889	0.938	0.914	0.829	0.963
(8,32)	0.879	0.939	0.909	0.820	0.960
(16,8)	0.891	0.951	0.921	0.845	0.970
(16,16)	0.898	0.939	0.918	0.838	0.968
(16,32)	0.901	0.943	0.922	0.846	0.967
(32,8)	0.905	0.942	0.923	0.848	0.971
(32,16)	0.893	0.947	0.920	0.842	0.970
(32,32)	0.892	0.950	0.921	0.844	0.970

### Ablation experiments

The analysis of feature information fusion presented in section Superiority of Feature Information Fusion highlights the significance of the evolutionary information component within the overall framework. This component comprises two CNN architectures alongside a channel attention mechanism. We conducted detailed ablation studies to evaluate the individual contributions of CNN1, CNN2, and the channel attention mechanism. Table 5 illustrates that the Sn of Opt_PredLLPS without CNN1 dropped from 90.5% to 89.9%, while the Sp rose from 94.2% to 94.6%. For Opt_PredLLPS without CNN2, Sn decreased slightly from 90.5% to 90.4%, with Sp increasing from 94.2% to 94.5%. Notably, Opt_PredLLPS lacking attention exhibited the most significant reduction in Sn, declining by 1.1%. This variation highlights the critical importance of the channel attention mechanism in the prediction of PS proteins and its ability to discern critical sequence information. In terms of Sn and AUC metrics, Opt_PredLLPS demonstrated a significant advantage over the two models lacking a CNN architecture. The utilization of multiple convolutional kernels enables the CNN component to capture diverse feature representations, thereby enhancing the model's expressiveness and overall performance.

**Table 5:** The performance of ablation experiment.

Model	Sn	Sp	Acc	MCC	AUC
Opt_PredLLPS	0.905	0.942	0.923	0.848	0.971
Without CNN1	0.899	0.946	0.923	0.846	0.969
Without CNN2	0.904	0.945	0.924	0.850	0.970
Without attention	0.894	0.946	0.920	0.843	0.970

### Comparison with other state-of-the-art methods on independent datasets

**Figure 5:**
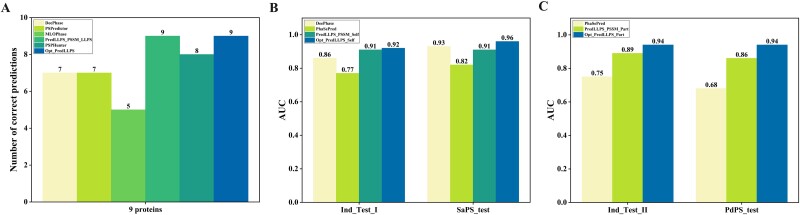
Comparison with other state-of-the-art methods on five independent datasets. (A) Performance of each model for nine proteins. (B) Performance of each model on Ind_Test_I and SaPS_test. (C) Performance of each model on Ind_Test_II and PdPS_test.

In this section, the proposed method is compared with six state-of-the-art methods: DeePhase, PhaSePred, PSPredictor, MLOPhase, PredLLPS_PSSM, and PSPHunter, employing various approaches. Initially, we evaluated each predictor on nine proteins given in [26], which are not included in all training datasets. The specific information of the nine sequences provided in the supplementary information. As shown in Fig. 5A, Opt_PredLLPS and PredLLPS_PSSM_LLPS successfully identified all nine proteins as LLPS proteins, while PSPHunter recognized eight, DeePhase and PSPredictor identified seven, and MLOPhase identified only five as LLPS proteins.

It has been noted that using different training and validation datasets to compare the performance of prediction tools can result in significant variability in results [57]. To mitigate bias, we applied the same training dataset of PS-Self proteins and PS-Part proteins used in [25] to build Opt_PredLLPS_Self model and Opt_PredLLPS_Part model, respectively. The superiority and stability of these two models were assessed on the Ind_Test_I, SaPS_test, Ind_Test_II, and PdPS_test datasets. The predictions result of the four independent tests are shown in Table S10–S13, and the AUC values are shown in Fig. 5B and C.

Opt_PredLLPS_Self achieved an AUC of 0.92 on Ind_Test_I and 0.96 on SaPS_test, surpassing the AUCs of all prior algorithms, including PredLLPS_PSSM_Self, which registered an AUC of 0.91 on these two datasets. Opt_PredLLPS_Part recorded an AUC of 0.94 on both the Ind_Test_II and PdPS_test datasets, outperforming the second-best AUC of PredLLPS_PSSM_Part by 0.05 and 0.08, respectively. Collectively, these results suggest that the modeling framework for distinguishing PS-Self proteins and PS-Part proteins from non-PS proteins is promising.

Next, these nine proteins were further distinguished as PS-Self or PS-Part proteins in the second task. The prediction results showed that eight out of nine proteins were predicted to be PS-Self proteins, and only one was predicted incorrectly. The detailed information of the nine proteins is shown in Table S14.

### Interpretable analysis of region importance

**Figure 6:**
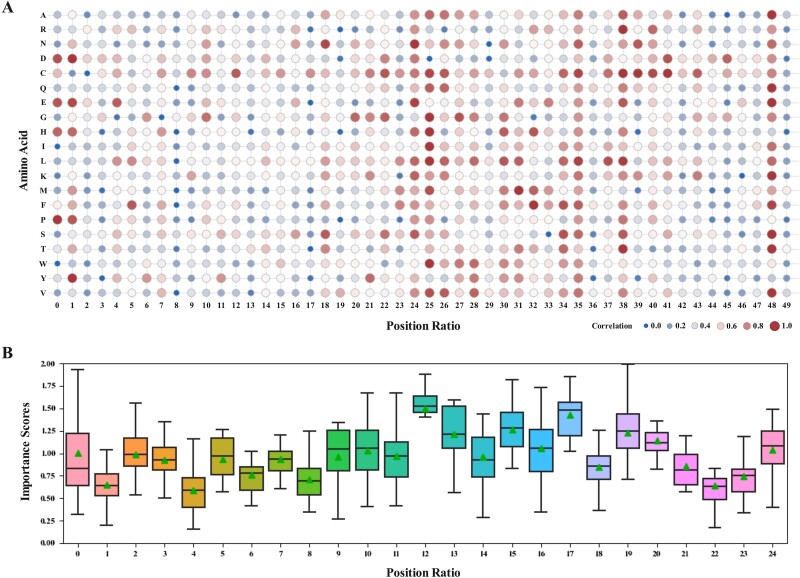
Interpretable analysis of region importance. (A) A correlation map between amino acid and position ratio. (B) A boxplot of the importance scores based on position ratio.

Due to the fact that the amino acid sequence is embedded with the characteristics related to phase separation, the sequence information of PS proteins was further explored using an ISM interpretation method [58]. For the nine protein sequences from PredLLPS_PSSM, each reference amino acid in each sequence was mutated to 1 of the other 19 alternative amino acids, and the probability was predicted using the second task model. The absolute difference between two output probabilities was used as the importance score to assess the impact of amino acid residues on the protein phase separation mechanism. Initially, we partition the sequence into 50 segments, and the total of the importance scores for each segment is termed the position ratio. By summing the importance scores of all segments, we derive a feature vector with a dimensionality of fifty for each sequence, thereby maintaining uniform feature dimensions across all sequences. [59]. In Fig. 6A, a complete correlation was established between the amino acid ratio and its position. The darker the color, the more important the impact of the amino acids at that position. Cysteine (C) exhibited the widest influence across the sequence, particularly affecting the central and tail regions. Glycine (G) had the most substantial effect on the central region, whereas proline (P) showed negligible impact on this area, possibly due to its ubiquity in PS proteins and the minimal effect of a single mutation on phase separation capability.

To further determine which region is more significant, each amino acid sequence was divided into 25 segments, and a combined score for each position was analyzed. It was evident from Fig. 6B that the importance score for the middle region was the highest, and the average value of its importance score is 1.5 times that of most other positions. Notably, the importance scores at position ratios 15, 17, and 19 were more pronounced than at adjacent positions, suggesting that these might be crucial areas of the phase-separated protein. This observation aligns with PSPHunter’s findings that most phase-separated proteins include three to four key regions. These insights may prove valuable for future studies on the LLPS mechanism and in identifying additional phase separation proteins.

## Conclusion

In this study, Opt_PredLLPS, a novel computational approach for identifying PS proteins and assessing their need for interaction with other macromolecules to undergo phase separation, is introduced. This model represents the first classification model that focuses on distinguishing between PS-Self and PS-Part proteins. Extensive benchmarking experiments conducted on five independent test datasets have shown that the combination of feature encoding and networking framework strategies is effective in elucidating the mechanism of LLPS separation. It is anticipated that Opt_PredLLPS will facilitate the accelerated discovery of both PS-Self and PS-Part proteins in future research.

**Key Points**

- We introduce a two-task predictor to discover potential phase-separated proteins and further evaluate its mechanism, named Opt_PredLLPS.
- The first task model of Opt_PredLLPS combines a convolutional neural network (CNN) and bidirectional long short-term memory (BiLSTM) through a fully connected layer, where the CNN utilizes evolutionary information features as input, and BiLSTM utilizes multimodal features as input. The second task model employs the XGBoost classification algorithm and 37 physicochemical properties, following a three-step feature selection.
- We confirm the effectiveness of Opt_PredLLPS on multiple benchmark datasets and use ISM to identify regions that play a key role in phase separation.

## Supplementary Material

Supporting_Information_bbae528

## Data Availability

The datasets and code used in this article are available at https://github.com/Zhou-Yetong/Opt_PredLLPS.
