# Supplementary material for: A two-task predictor for discovering phase separation proteins and their undergoing mechanism
Source: Brief Bioinform. 2024 Oct 21;25(6):bbae528. doi: 10.1093/bib/bbae528 (PMC11492799; doi:10.1093/bib/bbae528)
Supplement: Supporting_Information_bbae528 [file supporting_information_bbae528.docx]

Table S1. The 10-fold cross-validation results of different filters on CNN1.

| CNN1_filters | Sn | Sp | Acc | MCC | Pre | F1-score | AUC |
| --- | --- | --- | --- | --- | --- | --- | --- |
| 16 | 0.879 | 0.947 | 0.913 | 0.830 | 0.945 | 0.910 | 0.965 |
| 32 | 0.885 | 0.956 | 0.921 | 0.844 | 0.953 | 0.918 | 0.967 |
| 64 | 0.905 | 0.942 | 0.923 | 0.848 | 0.940 | 0.922 | 0.971 |

Table S2. The 10-fold cross-validation results of different filter on CNN2.

| CNN2_filters | Sn | Sp | Acc | MCC | Pre | F1-score | AUC |
| --- | --- | --- | --- | --- | --- | --- | --- |
| 16 | 0.895 | 0.941 | 0.918 | 0.838 | 0.939 | 0.916 | 0.969 |
| 32 | 0.905 | 0.942 | 0.923 | 0.848 | 0.940 | 0.922 | 0.971 |
| 64 | 0.893 | 0.947 | 0.920 | 0.843 | 0.945 | 0.918 | 0.968 |

Table S3. The 10-fold cross-validation results of different units on dense of CNN module.

| dense_units | Sn | Sp | Acc | MCC | Pre | F1-score | AUC |
| --- | --- | --- | --- | --- | --- | --- | --- |
| 8 | 0.896 | 0.954 | 0.925 | 0.852 | 0.952 | 0.922 | 0.967 |
| 16 | 0.891 | 0.951 | 0.921 | 0.845 | 0.949 | 0.918 | 0.970 |
| 32 | 0.905 | 0.942 | 0.923 | 0.848 | 0.940 | 0.922 | 0.971 |

Table S4. The 10-fold cross-validation results of different number of units of BiLSTM.

| BiLSTM_units | Sn | Sp | Acc | MCC | Pre | F1-score | AUC |
| --- | --- | --- | --- | --- | --- | --- | --- |
| 16 | 0.889 | 0.954 | 0.921 | 0.846 | 0.952 | 0.919 | 0.967 |
| 32 | 0.905 | 0.942 | 0.923 | 0.848 | 0.940 | 0.922 | 0.971 |
| 64 | 0.889 | 0.952 | 0.920 | 0.844 | 0.951 | 0.918 | 0.970 |

Table S5. The 10-fold cross-validation results of different units of dense of BiLSTM module.

| Dense_units | Sn | Sp | Acc | MCC | Pre | F1-score | AUC |
| --- | --- | --- | --- | --- | --- | --- | --- |
| 8 | 0.905 | 0.942 | 0.923 | 0.848 | 0.940 | 0.922 | 0.971 |
| 16 | 0.893 | 0.947 | 0.920 | 0.842 | 0.945 | 0.917 | 0.970 |
| 32 | 0.892 | 0.950 | 0.921 | 0.844 | 0.948 | 0.918 | 0.970 |

Table S6. The performance of AAIndex_AAC_35 combined with other features.

| Features | Sn | Sp | Acc | MCC | AUC |
| --- | --- | --- | --- | --- | --- |
| AAIndex_AAC_35 | 0.726 | 0.680 | 0.704 | 0.409 | 0.703 |
| AAIndex_AAC_35+ PS-related features | 0.687 | 0.663 | 0.675 | 0.353 | 0.675 |
| AAIndex_AAC_35+Sequence Length | 0.705 | 0.673 | 0.690 | 0.382 | 0.689 |
| AAIndex_AAC_35+IDR | 0.694 | 0.677 | 0.686 | 0.374 | 0.685 |
| AAIndex_AAC_35+LCR | 0.701 | 0.691 | 0.697 | 0.395 | 0.696 |
| AAIndex_AAC_35+ PLD-forming | 0.680 | 0.667 | 0.674 | 0.348 | 0.674 |
| AAIndex_AAC_35+ Granule propensity | 0.695 | 0.687 | 0.691 | 0.383 | 0.691 |
| AAIndex_AAC_35+FCR | 0.722 | 0.680 | 0.702 | 0.406 | 0.701 |
| AAIndex_AAC_35+NCPR | 0.712 | 0.677 | 0.695 | 0.391 | 0.694 |
| AAIndex_AAC_35+Omega | 0.726 | 0.677 | 0.702 | 0.405 | 0.701 |
| AAIndex_AAC_35+Kappa | 0.698 | 0.680 | 0.690 | 0.380 | 0.689 |
| AAIndex_AAC_35+PPII propensity | 0.705 | 0.680 | 0.693 | 0.388 | 0.693 |
| AAIndex_AAC_35+Hydrophobicity | 0.740 | 0.684 | 0.712 | 0.426 | 0.712 |
| AAIndex_AAC_35+Shannon Entropy | 0.705 | 0.684 | 0.695 | 0.391 | 0.695 |
| AAIndex_AAC_35+Hydrophobicity+Omega | 0.709 | 0.722 | 0.716 | 0.432 | 0.715 |
| AAIndex_AAC_35+Hydrophobicity+FCR | 0.712 | 0.652 | 0.683 | 0.367 | 0.682 |
| AAIndex_AAC_35+Hydrophobicity +FCR+ Omega | 0.712 | 0.645 | 0.679 | 0.361 | 0.679 |

Note: Since PScore cannot obtain the π-π interaction of some sequences, we do not use π-π interaction as a feature to train our model.

Table S7. The classification results of 37 Physicochemical Properties.

| Category | Accession number | Short description of the index |
| --- | --- | --- |
| alpha and turn propensities | CHOP780215 | Frequency of the 4th residue in turn |
|  | FASG760103 | Optical rotation |
|  | MAXF760106 | Normalized frequency of alpha region |
|  | QIAN880102 | Weights for alpha-helix at the window position of -5 |
|  | QIAN880112 | Weights for alpha-helix at the window position of 5 |
|  | QIAN880117 | Weights for beta-sheet at the window position of -3 |
|  | RICJ880117 | Relative preference value at C" |
| beta propensity | OOBM850104 | Optimized average non-bonded energy per atom |
|  | PALJ810112 | Normalized frequency of beta-sheet in alpha/beta class |
| Composition | NAKH920104 | AA composition of EXT2 of single-spanning proteins |
| Hydrophobicity | KLEP840101 | Net charge |
|  | ZIMJ680101 | Hydrophobicity |
|  | NAKH900106 | Normalized composition from animal |
|  | NAKH900113 | Ratio of average and computed composition |
|  | OOBM850103 | Optimized transfer energy parameter |
|  | PALJ810111 | Normalized frequency of beta-sheet in alpha+beta class |
|  | QIAN880128 | Weights for coil at the window position of -5 |
|  | RACS770103 | Side chain orientational preference |
|  | RACS820101 | Average relative fractional occurrence in A0(i) |
|  | RACS820103 | Average relative fractional occurrence in AL(i) |
|  | VASM830102 | Relative population of conformational state C |
|  | VASM830103 | Relative population of conformational state E |
|  | VELV850101 | Electron-ion interaction potential |
|  | Hydrophobicity | The hydrophobicity was calculated using the Kyte and Doolittle hydropathy scale |
| Physicochemical properties | OOBM770102 | Short and medium range non-bonded energy per atom |
|  | OOBM850102 | Optimized propensity to form reverse turn |
| Other properties | ISOY800108 | Normalized relative frequency of coil |
|  | RACS820107 | Average relative fractional occurrence in A0(i-1) |
|  | RICJ880101 | Relative preference value at N" |
|  | TANS770109 | Normalized frequency of coil |
| Unknown | CEDJ970102 | Composition of amino acids in anchored proteins (percent) |
|  | DIGM050101 | Hydrostatic pressure asymmetry index, PAI |
|  | GEOR030103 | Linker propensity from 2-linker dataset |
|  | KOEP990101 | Alpha-helix propensity derived from designed sequences |
|  | MONM990201 | Averaged turn propensities in a transmembrane helix |
|  | WILM950103 | Hydrophobicity coefficient in RP-HPLC, C4 with 0.1%TFA/MeCN/H2O |
|  | Omega | The mixing pattern of charged/proline residues and all other residues |

Table S8. The parameter setting of the six classification algorithms.

| Model | parameter | optimal parameter |
| --- | --- | --- |
| XGBoost | n_estimators: [80, 100, 120, 140, 160] | n_estimators=100 |
|  | learning_rate: [0.1, 0.2, 0.3, 0.4, 0.5] | learning_rate=0.3 |
|  | max_depth: [4, 6, 8, 10, 12] | max_depth=6 |
| LightGBM | num_leaves: [10, 15, 20, 25, 31] | num_leaves=25 |
|  | learning_rate: [0.01, 0.05, 0.1, 0.2, 0.3] | learning_rate=0.2 |
|  | max_depth: [3, 6, 9, 12, 15] | max_depth=15 |
| GBDT | n_estimators: [80,100,120,140,160] | n_estimators= 140 |
|  | learning_rate: [0.1,0.3,0.5,0.8,1] | learning_rate= 1 |
| SVM | C: 0.1~10 | C=1 |
|  | gamma: 0.1~10 | gamma= 1 |
| RF | n_estimators: 1~101, Step size is set to 5 | n_estimators=51 |
| ExtraTrees | n_estimators: 1~101, Step size is set to 5 | n_estimators=31 |

Note: Due to the small amount of data, the model parameters were not over-tuned to avoid overfitting.

Table S9. The performance of different models under optimal parameters.

| Model | Sn | Sp | Acc | MCC | AUC |
| --- | --- | --- | --- | --- | --- |
| XGBoost | 0.709 | 0.722 | 0.716 | 0.432 | 0.715 |
| RF | 0.708 | 0.684 | 0.696 | 0.395 | 0.696 |
| LightGBM | 0.684 | 0.705 | 0.695 | 0.391 | 0.695 |
| ExtraTrees | 0.703 | 0.638 | 0.670 | 0.344 | 0.670 |
| GBDT | 0.670 | 0.677 | 0.674 | 0.350 | 0.673 |
| SVM | 0.716 | 0.569 | 0.642 | 0.292 | 0.642 |

Table S10. The performance of each model on Ind_Test_I.

| Models | Sn | Sp | Acc | Pre | F1-score | AUC |
| --- | --- | --- | --- | --- | --- | --- |
| DeePhase | 0.73 | 0.99 | 0.92 | 0.95 | 0.82 | 0.86 |
| PhaSePred | 0.53 | 1 | 0.84 | 1 | 0.69 | 0.77 |
| PredLLPS_PSSM_Self | 0.85 | 0.96 | 0.93 | 0.87 | 0.86 | 0.91 |
| Opt_PredLLPS_Self | 0.81 | 0.97 | 0.93 | 0.91 | 0.86 | 0.92 |

Table S11. The performance of each model on SaPS_test.

| Models | Sn | Sp | Acc | Pre | F1-score | AUC |
| --- | --- | --- | --- | --- | --- | --- |
| DeePhase | 0.89 | 0.99 | 0.97 | 0.97 | 0.93 | 0.93 |
| PhaSePred | 0.63 | 1 | 0.86 | 1 | 0.78 | 0.82 |
| PredLLPS_PSSM_Self | 0.85 | 0.96 | 0.93 | 0.88 | 0.87 | 0.91 |
| Opt_PredLLPS_Self | 0.86 | 0.98 | 0.95 | 0.94 | 0.90 | 0.96 |

Table S12. The performance of each model on Ind_Test_II.

| Models | Sn | Sp | Acc | Pre | F1-score | AUC |
| --- | --- | --- | --- | --- | --- | --- |
| PhaSePred | 0.64 | 0.86 | 0.78 | 0.74 | 0.69 | 0.75 |
| PredLLPS_PSSM_Part | 0.81 | 0.96 | 0.93 | 0.88 | 0.84 | 0.89 |
| Opt_PredLLPS_Part | 0.83 | 0.96 | 0.93 | 0.87 | 0.85 | 0.94 |

Table S13. The performance of each model on PdPS_test.

| Models | Sn | Sp | Acc | Pre | F1-score | AUC |
| --- | --- | --- | --- | --- | --- | --- |
| PhaSePred | 0.49 | 0.87 | 0.71 | 0.75 | 0.6 | 0.68 |
| PredLLPS_PSSM_Part | 0.76 | 0.96 | 0.91 | 0.88 | 0.81 | 0.86 |
| Opt_PredLLPS_Part | 0.79 | 0.96 | 0.92 | 0.88 | 0.83 | 0.94 |

Note: The results for Tables S10-S13 are directly obtained from PredLLPS_PSSM. In order to facilitate comparison with previous models, the prediction results of our model are also retained to two decimal points.

Table S14. The specific information of 9 protein sequences.

| Protein description | Protein sequence |
| --- | --- |
| XP_028167223.1 calphotin-like [Ostrinia furnacalis] | MNSLVVLLSVVALAAASPSGLLLAPSAIVAPAAVSHQSRIDIQSSPAVVSTYASAPIATAAIATPAFYAAPAAIAAPALVGAHLIHKRSAPLIAAPALTSYVASPAITTYASAPIATAAIAAPGLALAAPTVIKSAIAAPVAYAAPAAIAAPAAVSHQSRIDIKSSPAVVSTYASAPIAAAAIAAPAVYPAPAAIAAPVAIAAPSVPLDTPEVIAARAAHFQAKALAGAHLIHKRSAPLIAAPALTTYVSSPAITTYSAAPVVHSAPLVQTYSSPILKANSVHPWSLSKHQSIFVHRSDNSTQTTMYKLVVLFSVVALAAAKPGLVAPLAYSAPVAYSAAVPATSSVSQYSSSVVHGSPVVPAVYSAPAVYSAPAVYSAPAVYSSSVVAPAVALAQAPHSPAVVLDAVNGVPLDTPEVVAARAAHYQAKALASHHLRKRSVGALSYSAVPVAHSVVAAPVSYSAPVVSSYSAAPVVSAYSSPVVSAYSTPVVSPYSSPVVSAYSAPVVASPVAYSAVVPKALSVHPW |
| XP_028168938.1 pro-resilin-like isoform X1 [Ostrinia furnacalis] | MILHVLWLSALAWSMSKCEPPVNSYLPPNAGNGGRPSSEYGPPGRNGGGGGSSGPGGVGRPSGGNNLNQPQDSYFPPGQSGSSPDTQYGPPSGRGNQGPQRPQFGSPSQNGQNGFGQGNGGNVPSSEYGTPGQNNRPGSSRQPSGQTPFGGDNRPQSSYGPPNGNQGFNSPSSGRGNGGPGSNNGRQGQRPESSYGPPPTGGFQAPGSGPSGRLDSQMPGSSYLSPGSDSSPGGFGGRQQSGGIPSSSYGTPGFGGSGASGRGFGIGNQGSDFGGDESDEPAKYEFSYDVDDPQTGTKFHHSEQRDGDVATGEYNVELPDGRKQVVEYEAGLQGYKPQIRYEGGSGGAGSGFGSGGSRGPQGGQGGGGQGYPRGGPGESFQGGNNDIGYSQGGPGQAGRPSRFEQANDFDQGSSAQLRGQESMGYPRGGPGNGGNGQGSRGYPGGEGGQDQSFGRPGSNGLESFNGSQDGEAGYPSGRPQGGDRNRPQGNGRGFPGGQGGGGGYPSGGPQGGRGPGGSGGDDDGYPSGGPSGPRGSGY |
| UUJ74911.1 cuticular protein [Trypoxylus dichotomus] | MFAKVFAIATFVATAQAGLIPAAPALSLGHAALAAPALSLGHAVGPALSLSHTALAAPAISLGHAVAAPALSLGHAAVAAPAYGIGHGLGLGYGLGHGAIAAPALVKAAPAIVKAAPAVDYVAYPKYEFNYGVSDAHTGDQKTQHEIRDGDVVKGSYSLHEADGTVRTVHYEADDHNGFNAVVTRSGHAAHPATPIAVAAPAKTIIAAPAIAHAAPVFAHAGPALAYGGLYGYKG |
| QWW34791.1 cuticular protein CPR12611 [Trypoxylus dichotomus] | MISSCGVALCYLALVSSVISAPVESGFSGVQYSNAQSLTPQQTQPSYITAPQPVQPQTPAYQPQSPSFGPPTQVVSQLLLPTNVDRQIQKAAQQIAGPQYVQQIQILQQQELANQAVAAAQRRAMRQRMLAQQAQQQHGGLSGLGGQFGAASSDVYSSQALAQAQTPQQYLQYQPQTARPVARPAPQVYSRPQPQGKDEDLEEYDTPASYKFGFDVTDDQNTNYQNRQEQRDGNKISGSYSVVDSDGFIRTVTYTADPKEGFKAEVVRKPTDIVVKIPTPTPAPKEQYVQNYSAPRGQESKGNVIYQYQ |
| 35753_Sequence from the transcriptome of Trypoxylus dichotomus | MKLLVLVACALAAVSADYHQPYYGPQHVPVIQNGVPVEPPEVQKARANHLAAFGGAPQYPAAPQYNAPAPQYNAPAPQYNAPAPQYNAPAPQYNAPAPYNQGPYNAPSQPVRQTPPAYQTSRVIPAIGNNGEPLDTPEVNAAKANHFAAYSQQHAALGQALAQAQPAGQGGYGGHGGHYRKRRSIYGGYGAYPQHVPQIAANGVPLDTPEVQHAKAAHLAAVAAASARVGAAGGAAGGAYDDGSYDGRYDNGAYDGRYDNGAYDGRYDDGSYDGRYDGAYDGRYDGGYGYGGGAWAGPIHIPVINANGVPVEPAAVQQARAAHLAAVGDAAARSGPGHIGYGHAAPWGAHY |
| XP_028171748.1 eukaryotic peptide chain release factor GTP-binding subunit-like [Ostrinia furnacalis] | MNGSTWILVCMVAIGVVAADVSHLGYNYKVPQTSYGVPSYQSAGSNYNTGYTQSSGSAGSSAGYQGLSAGQTVYQGHQGHQGHQGHQGSQGYTGYQVQGSAGNQGYSGYQGVNAASGASGISQYYPSVSSNGASSSSGSFVQPTYQVSGGSQGQSYQYQNQYQTSQQQKYQYQFQTQPAQIFKHFYVHEAPQEPEEAKPRQPVLLPPPQKHYKIIFIKTPSQAGGASQVVPVQQQNEEKTIVYVLVKKPEDYQDVVVPKIEQKPPSKPEVYFIKYKNKEDSQAVINNIVKDYDKGQNLVSISGADAGQSGSSAVYTGQVNAGAGESFVGHTGLTSGSTGSSSTGSFVSQGDASSSGGSVEYTGQQLVGSAQGISDGQLASVFTSVGQSENIGQQTSGLVFGSESSGGLNLATGHATGAGVVSSSYDSSASSTATYDNLNAISTSQGVPHETYGVPKFKEH |
| 35501_Sequence from the transcriptome of Locusta migratoria | MAFKLIVLAALVAVARAGYLGAPAVVAPGAPLAARAYAAPVAYAAPALRAAPLGYAAPALRAAPVAYAAPAVAKVAAPVAAAVPAAVAAEYDPHPQYSYSYDVQDALTGDSKTQQETRDGDVVQGSYSLVEPDGSIRTVDYTADPVNGFNAVVHKEAGAHPAPVVAKVAAPVAYAAPAIAKVAAPLAYAAPAYGKAILG |
| XP_028163720.1 larval cuticle protein LCP-30-like [Ostrinia furnacalis] | MRSLLVLCVTVSAVLAADSPFKPFQFGSNKFASNAGRYNVASASATASSSAGRYNPAAYDPSRYNPGRYVDNSGRYDPSKDNSGRYVPDGSGAYNGDRGDRGAAGGFYSGSSDKGGPGGFYSGSSDKGGPGGPGGAYKPEPNDGGKYKGDKSGGAYSGGASSGSGAGGFGSGSGAGAFGSGAGASAFGGASAGASASSGFGKGSGSGASKPVQYSGSAGSGTASGAGHYDYKFGIIRFENDVEPEGYHYLYETENKILAEEAGKVEKIDNENEGIRVKGFYEFVAPDGITYRVDYIADENGFQPSGAHLPK |
| XP_028160340.1 pupal cuticle protein G1A-like isoform X2 [Ostrinia furnacalis] | MFGKLMVFLCAAGIAAASEGYFYQAPASHGAAVVSTTAAPVYQSHVVATTAAPVYASAGAPTITTYSEPQVFHKSVSYQAAPAVSYAAQVVKQAPAVSYQTFSTPATYTASPVVFKSYAAPVSYAKSYAAPVVKSYAAAPAVSYAAAPVVKAYATAAPAVSYTAAPVVKTYATAAPAVSYSAAPAVSYSAAPVVKTYATAAPAVSYSAAPVVKTYATAAPAVSYSVAPAPLSYVKSYAAPAAVSYSAPAVSYSAPAAVSYSAPAVSYSAPAVSYSAPAVSYSAPAVSYSSYSAPAVEYGAPAYAYAAAPVHSVSYSSVPVSYVSYPGFAKKK |
